# Mental Health Literacy of Depression: Gender Differences and Attitudinal Antecedents in a Representative British Sample

**DOI:** 10.1371/journal.pone.0049779

**Published:** 2012-11-14

**Authors:** Viren Swami

**Affiliations:** Department of Psychology, University of Westminster, London, United Kingdom; University of Iowa Hospitals & Clinics, United States of America

## Abstract

**Background:**

Poor mental health literacy and negative attitudes toward individuals with mental health disorders may impede optimal help-seeking for symptoms of mental ill-health. The present study examined the ability to recognize cases of depression as a function of respondent and target gender, as well as individual psychological differences in attitudes toward persons with depression.

**Methods:**

In a representative British general population survey, the ability to correctly recognize vignettes of depression was assessed among 1,218 adults. Respondents also rated the vignettes along a number of attitudinal dimensions and completed measures of attitudes toward seeking psychological help, psychiatric skepticism, and anti-scientific attitudes.

**Results:**

There were significant differences in the ability to correctly identify cases of depression as a function of respondent and target gender. Respondents were more likely to indicate that a male vignette did not suffer from a mental health disorder compared to a female vignette, and women were more likely than men to indicate that the male vignette suffered from a mental health disorder. Attitudes toward persons with depression were associated with attitudes toward seeking psychological help, psychiatric skepticism, and anti-scientific attitudes.

**Conclusion:**

Initiatives that consider the impact of gender stereotypes as well as individual differences may enhance mental health literacy, which in turn is associated with improved help-seeking behaviors for symptoms of mental ill-health.

## Introduction

Despite the high prevalence of mental health disorders in Europe [1], about a half of cases do not receive any form of health care [2]. This is an important concern for public health because mental health disorders have considerable emotional, physical, and emotional consequences for individuals and their families [3]. Furthermore, under-utilization of health care services places a heavy socioeconomic burden on national economies [4]. Conversely, early help-seeking for symptoms of mental ill-health promotes opportunities for early intervention and is known to result in improved long-term outcomes [5]. Understanding the reasons for under-utilization of health care services for mental health disorders therefore remains an important topic of research.

In recent years, a number of factors have been found to act as barriers to optimal help-seeking, including economic hardship, limited access to psychiatric services, and lack of awareness of services [6]–[7]. Although these systemic factors are important, scholars have also recently focused on psychological factors that are associated with under-utilization of health care services, including negative attitudes toward professional help [8], cultural mistrust among ethnic minority groups [9], and skepticism of psychiatry as a science [10]. In addition, a growing body of research has focused on the lay theories and beliefs that non-professionals hold about health and illness, with the expectation that such psychological attitudes will influence when and how the general publics seek help for symptoms of ill-health [11]–[12].

One particular aspect of this literature is focused on ‘mental health literacy’, which refers to the knowledge and beliefs that the general public has about mental health disorders and their prevention [13]–[14]. Indeed, there is growing evidence that poor mental health literacy negatively impacts upon help-seeking behaviors for psychiatric symptoms and influences decisions about treatment and compliance [15]–[19]. Although the measurement of mental health literacy takes different forms, one of the most commonly-used methods involves the presentation of case vignettes that satisfy diagnostic criteria for mental health disorders [20]. Using this method, studies consistently show that members of the general public have difficulty identifying cases of mental health disorders.

For example, early work indicated that only about 40% of participants correctly identify vignettes of depression [13]. More recent evidence suggests that there may have been some improvement in this ability, at least in socioeconomically developed settings, with up to 70% of respondents now providing a correct label of depression [21]–[23]. Even so, the difficulty that the general public has in identifying symptoms of depression remains an important concern for public health for a number of reasons. Thus, failure to identify symptoms of depression is known to negatively impact upon help-seeking behaviors for symptoms of the disorder [21], [24], affects communication with health care practitioners [25]–[26], and may also be associated with negative emotional responses to patients of the disorder [27].

To date, however, the extant literature has tended to treat respondents as a fairly homogenous group and has not fully considered individual differences in mental health literacy. For example, despite scholarly discussions of ‘hegemonic masculinity’ as a barrier to help-seeking for mental health issues [28]–[29], previous work has not fully examined the impact of respondent and target gender on attitudes related to mental health. More specifically, the pursuit and endorsement of hegemonic masculinities has been identified as an important contributor to toxic health practices, through a denial of pain, weakness or vulnerability, the concealment of emotional and physical fragility, and a restriction of any help-seeking behavior that implies a loss of autonomy [28]–[32]. In this view, it is the very enactment of masculinity that may lead men to display more negative mental health literacy, which ensures that their behaviors conform to social convention.

In addition, there is some evidence that the ability to correctly identify psychiatric labels is influenced by respondents’ personality profiles and knowledge of psychiatry [33]–[34]. For example, previous work has reported that mental health literacy is impacted by such individual difference variables as the Big Five personality traits and psychiatric skepticism [33]–[34], but this has not been fully investigated, particularly among nationally representative samples. In short, then, the primary aim of this study was to examine the influence of respondent and target gender on the ability to recognize case vignettes of depression. In addition, this study also examined the extent to which mental health literacy of depression is influenced by individual psychological differences in attitudes toward psychological help, psychiatric skepticism, and anti-scientific attitudes. A secondary aim of the present study was to establish rates of mental health literacy in a nationally representative sample of British adults.

## Methods

### Ethical Statement

The ethics committee at the Department of Psychology, University of Westminster, specifically approved this study. All participants provided written informed consent.

### Participants and Procedure

In this survey, a sample of the general public in Britain, representative of demographic characteristics (gender, age, ethnicity) as reflected in the national census, was recruited. Eight research assistants used quota sampling (the non-probability equivalent of stratified sampling) to recruit a cohort of the general public. Satisfactory numbers for all quotas were achieved with 1,218 respondents, ranging in age from 18 to 78 years (*M*±*SD* = 47.32±15.53) (see Table 1 for additional demographics and representativeness of demographic quotas). Recruitment of participants took place through direct approaches in public locations in three cities (London, Birmingham, and Manchester) on weekdays between October 2011 and February 2012. Attempts were made to minimise selection bias by sampling at different times of the day and from a wide range of sites of congregate activities. Once participation had been agreed, participants provided informed consent and completed a paper-and-pencil survey in a quiet location. Completed surveys were returned to the researcher in a sealed envelope. All participants took part on a voluntary basis, were not remunerated for participation, and were verbally debriefed once they had returned their completed surveys.

**Table 1:** Demographic distribution of the study population (*N* = 1,218) and representativeness of demographic quotas.

Quota category	Quota	Percentage representativeof the UK generalpopulation (%)	Respondents in each quota	
			Percentage (%)	Number
Gender	Women	52	49.3	618
	Men	48	50.7	600
Age	18–24	11	7.5	91
	25–44	37	37.7	459
	45–64	31	32.9	401
	65+	21	21.9	267
Ethnicity	White	88	88.9	1083
	Asian	5	6.7	81
	Black	2	2.0	24
	Other	5	2.5	30

### Materials

#### Case vignettes

Respondents were randomly assigned to receive either a female (‘Kate’, *n* = 604) or male (‘Jack’, *n* = 614) version of a vignette describing a case meeting DSM-IV and ICD-10 diagnostic criteria for major depression [35]. The case vignettes describe individuals with symptoms of major depression, without use of clinical terminology, and are identical with the exception of the gender of the target (see Appendix 1):

For the past two weeks, Kate/Jack has been feeling really down. S/he wakes up in the morning with a flat, heavy feeling that stick with her/him all day. S/he isn’t enjoying things the way s/he normally would. In fact, nothing gives her/him pleasure. Even when good things happen, they don’t seem to make Kate/Jack happy. S/he pushed on through her/his days, but it is really hard. The smallest tasks are difficult to accomplish. S/he finds it hard to concentrate on anything. S/he feels out of energy and out of steam. And even though Kate/Jack feels tired, when night comes s/he can’t go to sleep. Kate/Jack feels pretty worthless and very discouraged. Kate/Jack’s family has noticed that s/he hasn’t been himself for the last month and that s/he has pulled away from them. Kate/Jack just doesn’t feel like talking.

Following presentation of the vignette, participants were asked if the individual described suffered from a mental health disorder (1 = *Yes*, 2 = *No*, 3 = *Not sure*). If participants returned an affirmative response, they were asked (using an open-ended question) to indicate the disorder. Responses were coded using maximal-response coding by three independent judges (inter-judge reliability = .95). Participants were also asked to rate, on a 7-point Likert-type scale (1 = *Not at all*, 7 = *Extremely*) how distressing they believed the condition was, how difficult it would be to treat the condition, and how sympathetic they were to the person described in the vignettes. Finally, they were asked to indicate, assuming they were friends, how likely they were to suggest that the person described seek help (1 = *Not at all*, 7 = *Definitely*).

#### Attitudes towards professional help

General attitudes toward seeking professional help for mental health issues were measured using the Attitudes Towards Seeking Professional Psychology Help Scale [36]. This scale consists of 29 items rated on a 4-point Likert-type scale (0 = *Disagree*, 3 = *Agree*). Although the scale consists of several subscales, factorial studies indicate the underlying structure of the scale is unstable [36]. It is recommended that researchers use the overall mean [36], with higher scores indicating more negative attitudes toward seeking professional help for mental health issues. In the present study, internal consistency for this scale was acceptable (Cronbach’s α = .79).

#### Psychiatric skepticism

Psychiatric skepticism was assessed using the Psychiatric Skepticism Scale [10], [33], a 16-item measure of an individual’s degree of skepticism towards psychiatry as a legitimate science. Items were rated on a 7-point Likert-type scale (1 = *Strongly disagree*, 7 = *Strongly agree*). Factorial work has shown that the scale has a one-dimensional structure and good indices of internal reliability and validity [10]. An overall score was computed as the mean of all items, with higher scores indicating greater psychiatric skepticism. In the present study, internal consistency for this scale was very good (Cronbach’s α = .92).

#### Attitudes toward science

Negative attitudes toward contemporary science were measured using the 8-item Anti-Scientific Subscale of the New Age Beliefs Scale [37]. Items were rated on a 7-point Likert-type scale (−3 = *Totally unbelievable*, +3 = *Totally believable*) and an overall score was computed as the mean of all items (higher scores reflect greater anti-scientific attitudes). Previous work has shown that this subscale has a one-dimensional structure with adequate internal consistency and good patterns of validity [37]. In the present study, this scale had good internal consistency (Cronbach’s α = .75).

### Statistical Analysis

To examine differences in responses to the case vignette as a function of respondent and target gender, chi-square tests were used. Phi (φ) provides a measure of effect size, such that φ^2^ gives an estimate of the proportion of variance that is common to the two variables entered in the analysis. Differences as a function of target and respondent gender on ratings of distress, difficulty of treatment, sympathy, and likelihood of recommending help were analyzed using 2×2 analyses of variance (ANOVAs) with follow-up tests of simple effects for significant results. Partial eta-squared (η_p_^2^) provides a measure of effect size for ANOVA results, while Cohen’s *d* was used to examine the effect sizes of *t*-tests. Finally, to examine relationships between attitudes toward depression and individual psychological differences, bivariate correlations between all variables were calculated using aggregated data across respondent and vignette gender. To examine predictors of ratings, multiple linear regressions were computed with the ratings as dependent variables and attitudes toward seeking psychological help, psychiatric skepticism, and anti-scientific attitudes as predictor variables.

## Results

### Preliminary Analyses

An independent samples *t*-test indicated that there was no significant difference between women and men in mean age, *t*(1216) = 1.86, *p* = .064, *d* = 0.11. There was also no significant difference between women and men in the distribution of ethnic groups, χ^2^(3) = 7.64, *p* = .054, φ = .07.

### Symptom Recognition

Responses to the question of whether the individuals described in the vignettes suffered from a mental health disorder were examined first. For the female vignette, 56.8% of respondents said she did, 10.1% said she did not, and 33.1% were unsure. Of those who said she did, the most frequent follow-up response was ‘depression’ (77.1%). For the male vignette, 52.0% of participants indicated that the person described had a mental health disorder, 20.8% said he did not, and 27.2% were unsure. Of those who reported that he did have a disorder, the most frequent response was ‘depression’ (84.4%). There was a significant difference in responses to the question of whether the described individual suffered from a mental health disorder as a function of the gender of the described individual, χ^2^(2) = 27.51, *p*<.001, φ = .15. Respondents were more likely to indicate that the male vignette did not suffer from a disorder compared to the female vignette. Participant gender did not have a significant influence on responses for the female vignette, χ^2^(2) = 4.17, *p* = .124, φ = .12. On the other hand, men were more likely than women to indicate that the male vignette did not suffer from a mental health disorder, χ^2^(2) = 8.78, *p* = .012, φ = .12.

### Attitudes Toward Depression

Next, a series of 2×2 ANOVAs were conducted, with ratings of distress, difficulty of treatment, sympathy, and likelihood of recommending help as dependent variables, and vignette gender and respondent gender, respectively, as independent variables. As can be seen in Table 2, for ratings of distress, there was a significant interaction between target and respondent gender. Women rated the male case vignette as being significantly more distressing than did men, *t*(612) = 4.54, *p*<.001, *d* = 0.37, but there was no significant difference between women and men’s ratings of distress for the female vignette, *t*(602) = 0.42, *p* = .674, *d* = 0.03.

**Table 2:** Descriptive statistics and results of the analysis of variance examining the impact of gender (vignette and respondent) on attitudes toward cases of depression.

Item	Vignette gender		Respondent gender		Main effect of vignette gender			Main effect of respondent gender			Interaction between vignette and respondent gender		
	Female	Male	Female	Male	*F*	*p*	η_p_^2^	*F*	*p*	η_p_^2^	*F*	*p*	η_p_^2^
Distress	5.08 (1.30)	4.80 (1.60)	5.08 (1.44)	4.81 (1.41)	11.09	.001	<.01	10.93	.001	<.01	14.62	<.001	.01
Difficulty of treatment	4.19 (1.52)	3.78 (1.62)	3.99 (1.55)	3.98 (1.62)	20.16	<.001	.02	0.01	.910	<.01	1.11	.293	<.01
Sympathy	5.18 (1.50)	4.83 (1.79)	4.98 (1.56)	5.03 (1.75)	13.70	<.001	.01	0.21	.647	<.01	4.93	.027	<.01
Likelihood of recommending help	5.65 (1.48)	5.25 (1.87)	5.43 (1.81)	5.47 (1.58)	16.75	<.001	.01	0.22	.642	<.01	9.32	.002	<.01

For the difficulty of treatment item, there was a significant main effect of target gender, with respondents believing that the female vignette would be more difficult to treat than the male vignette. In terms of sympathy ratings, there was a significant target gender by respondent gender interaction. Women were more sympathetic toward the male vignette than men, *t*(612) = 1.76, *p* = .042, *d* = 0.14, but there was no significant difference between women and men’s sympathy for the female vignette. Finally, there was also significant interaction for ratings of likelihood of recommending help. Men were more likely to recommend that the female target seek help than women, *t*(602) = 2.83, *p* = .005, *d* = 0.23, but there was no significant difference between women and men in their likelihood of suggesting that the male target seek help, *t*(612) = 1.66, *p* = .098, *d* = 0.13.

### Inter-Scale Correlations

To examine associations between attitudes toward depression and individual psychological differences, bivariate correlations between all variables were calculated using aggregated data across respondent and vignette gender (see Table 3). Results showed consistent small-to-moderate correlations between attitudes toward seeking psychological help and ratings of distress, difficulty of treatment, sympathy, and likelihood of recommending help. Consistent correlations were also found between psychiatric skepticism and anti-scientific attitudes, respectively, and the ratings of the vignettes. To examine predictors of the latter ratings, multiple linear regressions were computed with the ratings as dependent variables and attitudes toward seeking psychological help, psychiatric skepticism, and anti-scientific attitudes as predictor variables (see Table 4).

**Table 3:** Descriptive statistics and inter-scale correlations between attitudes toward cases of depression and remaining variables included in the present study.

	(1)	(2)	(3)	(4)	(5)	(6)	(7)
(1) Distress		.33	.64	.64	−.18	−.22	−.28**
(2) Difficulty of treatment			.36	.26	.14	.19	.09**
(3) Sympathy				.69	−.16	−.14	−.12**
(4) Likelihood of recommending help					−.31	−.28	−.27**
(5) Attitudes toward seeking professional help						.40	.45**
(6) Psychiatric skepticism							.55**
(7) Anti-scientific attitudes							
*M*	4.94	3.98	5.01	5.45	1.98	3.89	0.03
*SD*	1.43	1.59	1.66	1.70	0.41	0.92	1.27

Note: *N* = 1218. All correlations significant (*p*<.001).

**Table 4:** Individual psychological differences as predictors of ratings of case vignettes of depression.

Dependent variable	Independent variable	*B*	*SE*	β	*t*	*p*
Distress	Attitudes toward seeking professional help	−.19	.11	−.05	1.70	.045
	Psychiatric skepticism	−.13	.05	−.08	−2.43	.008
	Anti-scientific attitudes	−.23	.04	−.21	−6.02	<.001
Difficulty of treatment	Attitudes toward seeking professional help	.36	.12	.09	2.93	<.001
	Psychiatric skepticism	.29	.06	.17	4.81	<.001
	Anti-scientific attitudes	−.06	.04	−.05	−1.35	.089
Sympathy	Attitudes toward seeking professional help	−.45	.13	−.11	−3.49	<.001
	Psychiatric skepticism	−.14	.06	−.08	−2.18	.015
	Anti-scientific attitudes	−.04	.05	−.03	−0.80	.212
Likelihood of recommending help	Attitudes toward seeking professional help	−.83	.13	−.20	−6.60	<.001
	Psychiatric skepticism	−.25	.06	−.14	−4.10	<.001
	Anti-scientific attitudes	−.14	.05	−.11	−3.13	<.001

The regression with ratings of distress was significant, *F*(3, 1214) = 37.60, *p*<.001, Adj. *R* ^2^ = .10, with more negative attitudes toward seeking psychological help, greater psychiatric skepticism, and greater anti-scientific attitudes predicting lower ratings of distress. The regression with ratings of treatment difficulty was also significant, *F*(3, 1214) = 16.21, *p*<.001, Adj. *R* ^2^ = .06, with more negative attitudes toward seeking psychological help and greater psychiatric skepticism predicting a stronger belief that depression would be difficult to treat. The third regression was also significant, *F*(3, 1214) = 15.17, *p*<.001, Adj. *R* ^2^ = .05, with more negative attitudes toward seeking psychological help and greater psychiatric skepticism predicting lower sympathy. The final regression was significant, *F*(3, 1214) = 59.12, *p*<.001, Adj. *R* ^2^ = .18, with more negative attitudes toward seeking psychological help, greater psychiatric skepticism, and greater anti-scientific attitudes all predicting lower likelihood of respondents recommending help.

## Discussion

Based on a large survey of the British population, the present study provides evidence that there are individual differences in mental health literacy and attitudes toward depression. First, results showed that there were gender differences in mental health literacy as a function of both the target and the respondent. Specifically, respondents were more likely to indicate that the male vignette did not suffer from a disorder compared to the female vignette, and men were more likely than women to indicate that the male vignette did not suffer from a mental health disorder. In addition, respondents – particularly men – rated the case of the female vignette as significantly more distressing, difficult to treat, and deserving of sympathy than they did the case of the male vignette.

These findings are consistent with the notion that dominant gender role ideologies shape attitudes toward mental health [28]–[32]. To the extent that mental illness is inconsistent with notions of hegemonic masculinity that stress toughness and strength [30], respondents may be less likely to view men with symptoms of depression as suffering from a mental health disorder and, consequently, may adopt less positive attitudes toward such persons. In this view, hegemonic masculinities leave men with few resources with which to construct healthy attitudes toward mental health behaviors [28]–[30]. The ways in which men relate to dominant forms of masculinity thus appear to impact on their mental health-related conceptions and attitudes. In turn, such beliefs may affect treatment-seeking recommendations: respondents were also more likely to suggest that the female vignette seek help compared with the male vignette.

The present results further suggest that community respondents should not be treated as a homogenous group; rather, there appear to be important individual psychological differences in responses to cases of depression. Specifically, this study showed that more negative attitudes toward psychological help, psychiatry, or science, respectively, were significant predictors of attitudes toward vignettes of persons suffering from depression. While most previous studies have focused on mental health literacy (e.g., symptom recognition) as a means of promoting optimal help-seeking [38], the present work suggests that there may be some utility in focusing on broader individual differences. For example, intervention programs aimed at promoting more positive attitudes toward psychiatry and better scientific literacy may result in improved mental health literacy and, in turn, optimal help-seeking behaviors.

Limitations of the present study include the fact that only vignettes of depression were utilized, meaning that findings may not generalize to other mental health disorders. In a similar vein, the variance explained by the regression models was small, suggesting that there may be other predictors that were not accounted for. The latter may include knowledge of mental health disorders, perceived availability and quality of services, previous experience with healthcare providers, gender role orientation, and personality variables. Indeed, in future work it will be important to incorporate frameworks of analysis that take concurrently take into account the myriad of factors, both structural and psychological, that have been shown to impact upon mental health literacy. Third, the use of quota sample, rather than a random sample, may have introduced substantial sampling biases as selection of participants is determined by the researcher. Finally, questions remain as to the ecological validity of the present design: although the methodology used here is widely-used in the literature on mental health literacy, the extent to which responses to case vignettes mirror behavior in real-life situations remains unclear.

These limitations notwithstanding, the present results underscore the role of individual differences in mental health literacy. Initiatives that consider the impact of gender stereotypes as well as individual differences may enhance mental health literacy, which in turn is associated with improved help-seeking behaviors for symptoms of mental ill-health. In the future, it will be important to more carefully assess inter-individual differences in mental health literacy as a function of both the target and the respondent. The evidence presented here suggests that treating community individuals from the same cultural context as a homogenous group may in fact mask marked inter-individual differences, which affect mental health literacy.
